# Open sesame: unlocking mechanics of clots formed under flow

**DOI:** 10.1016/j.rpth.2026.106935

**Published:** 2026-08-31

**Authors:** Timea Feller

**Affiliations:** Discovery and Translational Science Department, Leeds Institute of Cardiovascular and Metabolic Medicine, University of Leeds, Leeds, UK

Blood clot formation is a complex process governed by the interplay of coagulation factors, cellular components, blood flow, and platelet-driven clot contraction, all of which can profoundly influence clinical outcomes. Important steps are being taken to detangle this complexity. Eyisoylu et al. [1] take a step forward by showing not only how increasing flow rates shape thrombus formation and final composition but also how these changes translate into the mechanical properties of the formed clot. Investigating the effect of flow on clot structure, and especially on clot mechanics, is technically challenging. To address this, the authors modified a microfluidic-on-chip model making it openable by sealing it with vacuum. This innovation allowed direct mechanical characterization of clots formed under controlled flow conditions within 1 experimental workflow.

Eyisoylu et al. [1] followed clot formation with confocal microscopy in real time. This provided clear evidence that higher shear rates accelerate and enhance platelet aggregation. While the onset of platelet aggregate formation was shear dependent, fibrin network formed to stabilize these aggregates at a relatively constant time point, 6 to 8 minutes after initiation. Essentially, platelet deposition gets well ahead of fibrin stabilization as shear rates increased. This becomes critical for pathologic shear rates (>3000/s), where von Willebrand factor begins to anchor platelets extensively, leading to early formation of larger and more numerous platelet islands that are often embolized before the fibrin network could form and stabilize them. Taken together, these observations strongly support the conclusion that enhanced platelet deposition underlies the formation of the higher, more occlusive thrombi observed at pathologic shear rates.

Mechanical testing was then performed in combination with fluorescent microscopy, adding information about the local composition of mature clots to the measured indentation stiffness. Interestingly, when clots formed under pathologic shear were compared with those formed under physiological shear, stiffness differed markedly only in platelet- and fibrin-rich regions, while remaining very similar in red blood cell (RBC)- and fibrin-rich regions or regions composed predominantly of RBCs. Altogether, Eyisoylu et al. [1] demonstrated that pathologically high shear rates lead to platelet-rich clots that are significantly stiffer than clots formed under physiological shear rates, highlighting the dominant role of platelet-driven stiffening.

Previous studies have already shown that platelet-rich structures form before fibrin stabilization under arterial shear conditions [2,3]. However, this study provides clear evidence that islands of platelets that are formed too rapidly in advance of fibrin network stabilization occasionally break off before the fibrin network could provide clot integrity. Importantly, the authors show that this early embolization only happens under pathologic shear conditions. A further strength of the study is the use of whole blood flowing over a collagen-rich surface, providing a more physiologically relevant model of atherosclerotic plaque rupture than some of the earlier experimental systems.

One of the most significant additions of this study is that the vacuum-sealed flow chamber can be opened, allowing subsequent mechanical testing of the same blood clots that were formed and imaged under flow (Figure). While many studies have provided detailed mechanical characterization of clots, including fracture behavior, they could not account for the effects of flow [4,5]. Conversely, when clots are formed under flow, measuring mechanical properties would be challenging without perturbing the formed clot [6,7]. This includes an earlier study from Eyisoylu et al. [8], where clots formed in a Chandler loop were tested under compression but the formed clots had to be cut to a standard size before testing. In the recent study, mechanical testing was done *in situ*.

**Figure:**
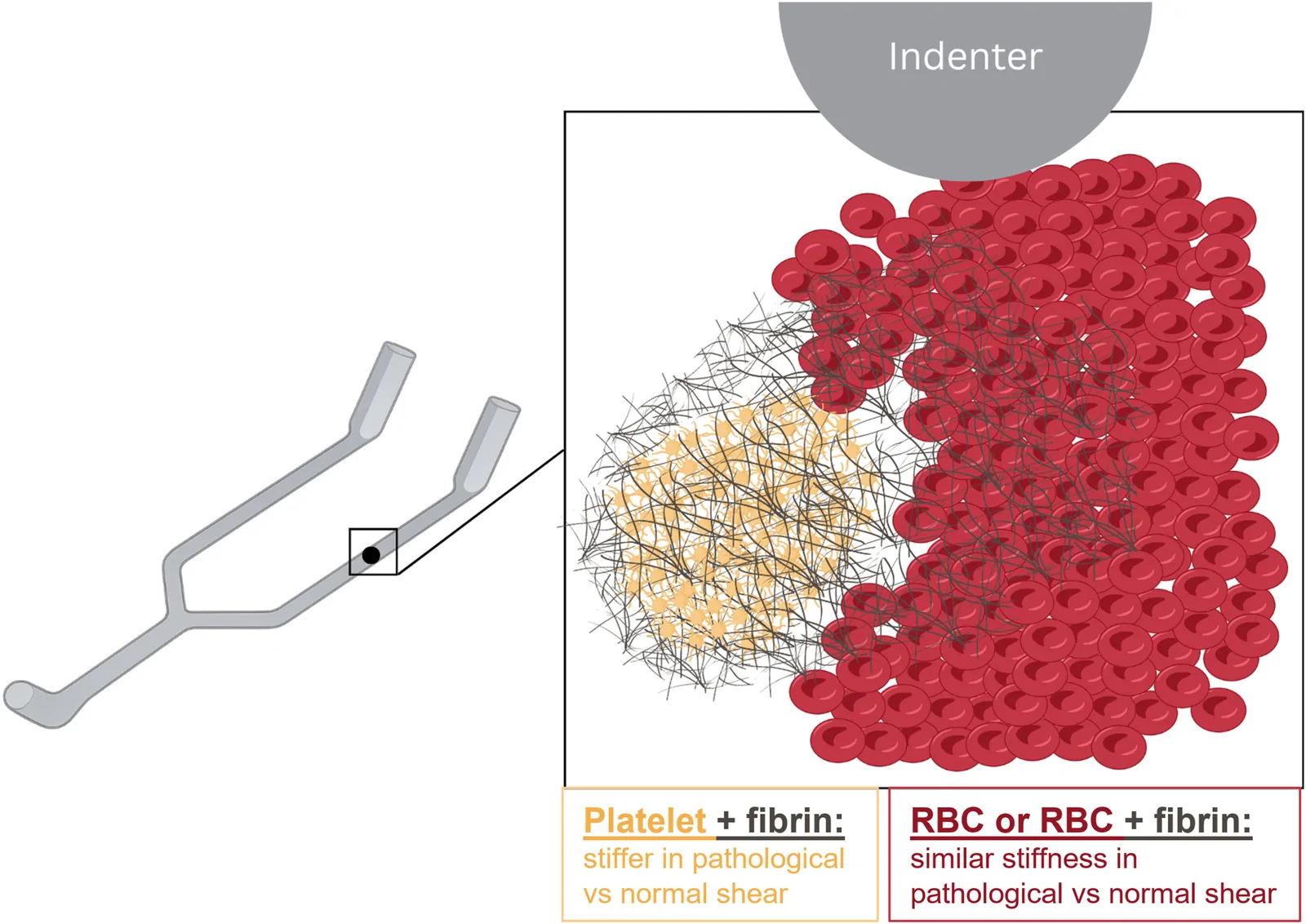
A vacuum-sealed flow chamber with parallel flow channels (left) enables *in situ* mechanical testing (inset) of blood clots formed under flow. Dark spot on flow channel represents location where thrombus formation was initiated by collagen and tissue factor coating. Microindentation revealed the importance of investigating local mechanical properties as clot stiffness significantly increased at high shear rates for platelet–fibrin-rich regions but not for red blood cell–rich regions.

The device builds on 2 existing concepts: parallel flow channel design developed by Berry et al. [9] and an openable single-channel flow system developed by Swieringa et al. [10] for correlative structural and mechanical analysis. In the reported study, these concepts are combined in an openable parallel-channel device. This design allows clot growth in the vertical direction, resulting in clot geometries that are closer to physiological conditions. Specifically, the parallel channels allow an occlusive clot to develop in 1 channel while maintaining flow through the adjacent channel. Similarly, the slightly decreased width-to-height ratio (500 μm:70 μm ≈ 7) provides sufficient chamber height for vertical clot growth while remaining within recommended design guidelines [11]. By contrast, previous openable flow-chamber designs often used width-to-height ratios of >10 to ensure a uniform shear-stress distribution [12] but opening the possibility to have restricted clot growth in the vertical direction.

The sealable vacuum chamber opens new opportunities for mechanical characterization of clots, which is highly relevant for understanding embolization. The increased local stiffness observed in platelet- and fibrin-rich regions of clots formed under pathologic shear is consistent with platelet contraction stiffening the clot by pulling on fibrin fibers [13]. Consequently, since fibrin forms the structural backbone of mature clots, understanding how pathologic shear affects fibrin rupture behavior may be more informative for predicting clot stability than stiffness measurements alone. Previous study has shown that embolization frequency increases when the toughness of individual fibrin fibers is reduced [14]. By contrast, the indentation measurements used in the reported study with a penetration depth of 2 μm primarily probe low-strain properties of fibrin, which does not necessarily predict rupture behavior or toughness [15]. The key question therefore remains whether the increased stiffness of clots formed under pathologic shear reflects enhanced stability by producing tougher fibrin assemblies or reflects increased embolization risk by indicating the presence of prestrained fibrin fibers.

Further high-resolution imaging would complement the mechanical measurements by revealing structural changes in the fibrin network, such as fiber thickness and pore size, similar as in previous work by the de Vries et al. [16]. It would also be interesting to determine distribution of polyhedral erythrocytes, a compressed form of RBCs [17], within the clot and how their presence correlates with local mechanical properties. In this context, it is notable that regions containing only RBCs were not significantly stiffer in clots formed under pathologic shear than in those formed under physiological shear, despite their overall higher platelet content. Altogether, such structural analyses would help to identify local weak points within the clot that may contribute to embolization. Combined with investigations of clot lysis, they would provide valuable structural context alongside the mechanical measurements presented in the reported study.

Overall, this study provides an elegant platform that addresses the gap between physiologically relevant clot formation under flow and *in situ* mechanical characterization. By combining an openable chamber design with parallel flow channels, the authors create new opportunities to link clot structure, mechanics, stability, and ultimately embolization risk within the same experimental system. As such, this work represents an important methodological advance that is likely to support future studies linking both mechanical and structural properties of thrombi to their function in health and disease.
